# Testing for Similarity of Dose Response in Multiregional Clinical Trials

**DOI:** 10.1002/sim.70255

**Published:** 2025-09-08

**Authors:** Holger Dette, Lukas Koletzko, Frank Bretz

**Affiliations:** ^1^ Department of Mathematics Ruhr‐Universität Bochum Bochum Germany; ^2^ Statistical Methodology Novartis Pharma AG Basel Switzerland; ^3^ Section for Medical Statistics, Center for Medical Statistics, Informatics, and Intelligent Systems Medical University of Vienna Vienna Austria

**Keywords:** constrained bootstrap, equivalence testing of curves, subgroup analysis

## Abstract

This article addresses the problem of determining whether the dose response relationships between subgroups and the full population in a multiregional trial are similar. Similarity is assessed in terms of the maximal deviation between the dose response curves. We consider a parametric framework and develop two powerful bootstrap tests: one for assessing the similarity between the dose response curves of a single subgroup and that of the full population, and another for comparing the dose response curves of multiple subgroups with that of the full population. We prove the validity of these tests, investigate their finite sample properties through a simulation study and illustrate the methodology with a case study.

## 1 Introduction

A multiregional clinical trial is a single trial carried out simultaneously across various regions under a common protocol to investigate the effect of an investigational drug. Its primary goal is often to draw conclusions about the drug's effect across all the regions participating in the trial. Conducted within the framework of a global drug development program, a multiregional clinical trial aimed at bridging purposes can facilitate the drug's registration across all involved regions. In recent years, such trials have received increasing attention for their potential to reduce resources by avoiding the need for multiple, regional trials [1]. Accordingly, multiregional clinical trials typically have at least two main objectives: demonstrating the drug's efficacy within individual regions and comparing trial results across regions to confirm that the drug's effects are not influenced by ethnic or other regional factors.

The guideline by the International Council of Harmonisation (ICH) on general principles for planning and design of multiregional clinical trials highlights the importance of identifying intrinsic and/or extrinsic ethnic factors that could impact drug responses in the early stages of drug development, prior to the design of confirmatory multiregional studies [1]. Consequently, early‐phase trials are increasingly being conducted across multiple countries or regions, especially those focusing on dose response [2]. The primary aim of multiregional dose response trials is to establish the dose response relationship using data from the full (i.e., global) population. Once this primary goal is achieved, it becomes important to evaluate whether the results applicable to the full population can be reliably extended to specific regions. To this end, the dose response relationship observed in any given region should be similar to that of the full population. If discrepancies are observed, further investigation into the intrinsic and/or extrinsic ethnic factors affecting these outcomes may be necessary.

Many authors consider the problem of how to choose the subgroup sample sizes in multiregional confirmatory trials in order to allow observing a consistent treatment effect between a regional subgroup and the full population with acceptable probability [3, 4, 5]. More recently, the problem of sample size allocation for demonstrating consistency (or similarity) in multi‐regional dose finding trials is addressed as well [6, 7]. In particular, consistency between dose response profiles of a regional subgroup and the global population is measured by the probability that the maximal deviation between the two curves falls below a fixed threshold [6]. More precisely, the authors measure consistency using the probability

$$\begin{array}{l} \eta_{m}(n_{1})=\mathbb{P}({\hat{d}}_{\infty }<\delta |\mu =\mu_{m}) \end{array}$$

where δ>0 is a similarity threshold, $n_{1}$ denotes the sample size corresponding to the subgroup, $\mu_{m}$ is a candidate dose response model and ${\hat{d}}_{\infty }$ is an estimate of the maximum deviation between the dose response curves of the target subgroup and the population over the dose range of the trial. Under the assumption of identical curves for subgroup and population the authors consider the problem of how to minimally choose $n_{1}$ in order to guarantee $\eta_{m}(n_{1})\geq \eta^{*}$ while accounting for model uncertainty, where $\eta^{*}\geq 0$ denotes a fixed probability. The challenge of this approach is the determination of the probability $\eta_{m}(n_{1})$. In order to facilitate these sample size calculations, the calculation of the maximum deviation ${\hat{d}}_{\infty }$ is restricted to the used dose levels, say, $d_{1},\ldots ,d_{r}$, and an approximation formula for the proposed consistency probability is derived by exploiting the asymptotic normality of the vector of curve differences at those dose levels [7]. Equivalence tests pursue a similar approach and are a common tool to decide whether parameters of interest such as the area under the curve or the peak concentration of two groups are similar. Here, the null hypothesis is defined as an effect exceeding a given threshold and under the alternative the effect is smaller than this bound. Meanwhile there exists well developed methodology on testing the equivalence of finite dimensional parameters [8]. While a large body of this literature refers to applications in medicine, in particular pharmacokinetics, equivalence tests have also been used in other areas such as economics, psychology or biology [9, 10, 11]. A common feature of all these references consists in the fact that equivalence refers to finite dimensional (often one‐) dimensional parameters which are considered similar if the distance between them, measured by some metric, is smaller than a fixed threshold.

However, the problem of investigating the similarity between infinite‐dimensional parameters, such as real‐valued curves over a continuous covariate space, is far less explored. In the literature, tests for the similarity of quantile curves and power law distributions are proposed [12, 13] and in the context of drug development tests for the similarity between dose response curves of two distinct groups of patients and confidence bands for the difference between the two curves are developed utilizing a parametric model assumption [14, 15, 16, 17]. More recently, more powerful similarity tests based on constrained parametric bootstrap are proposed [18, 19, 20] as well as a Bayesian approach [21] to the problem. A common feature in all these references consists in the fact that parameters or curves corresponding to two different and independent groups are compared.

In this paper, we consider the problem of assessing whether the dose response relationships of one or more (regional) subgroups and the full population are similar from a hypothesis testing point of view. Similar to much of the aforementioned literature, we define a parametric model for the clinical trial data and formulate appropriate statistical hypotheses that capture the similarity problem. We propose two powerful parametric bootstrap tests: one for assessing the similarity between the dose response curves of a single subgroup and that of the full population, and another for comparing the dose response curves of multiple subgroups with that of the full population. We prove the validity of these procedures and investigate their performance across various scenarios by means of a simulation study which includes small sample sizes. Finally, we illustrate the use of these tests in a multiregional dose finding case study. Our work differs from the literature [6, 7] with respect to several aspects. First, the dose response functions of the subgroups in our model are not restricted to be identical. In fact, they are allowed to have different parametric forms. Second, our model allows not only for two, but also for more than two subgroups and we also propose a test for simultaneously assessing the similarity between several subgroups and the full population. Third, we compare the dose response curve on the full dose range and not only at the dose levels used in the clinical trial. Fourth [6, 7], do not address the problem of similarity of the dose response relationships between subgroups and the full population by means of hypothesis testing, while in this paper we define similarity and nonsimilarity in terms of statistical hypotheses and develop a constrained bootstrap approach for testing these hypotheses.

The remainder of this paper is organized as follows. In Section 2 we introduce the framework and methodology for assessing similarity of one or several subgroups with the full population. Section 3 and Section 4 are dedicated to a simulation study and numerical example, respectively. We conclude with a discussion in Section 5 and defer all mathematical details to the Supporting Information.

## 2 Assessing Similarity of Subgroups With the Full Population

Suppose a dose response trial is conducted with r dose levels $d_{1},\ldots ,d_{r}$ in the dose range $𝒟=[d_{1},d_{r}]$, where $d_{1}=0$ denotes the placebo group. Assume that a population of patients can be decomposed into k disjoint subgroups (corresponding to the different regions in a multiregional clinical trial). Let $n_{\ell }$ denote the number of patients belonging to subgroup ℓ=1,…,k, and denote by $n=n_{1}+\ldots +n_{k}$ the total number of patients recruited for the trial. Each patient is randomized to one of the dose levels $d_{1},\ldots ,d_{r}$. Let $n_{\ell ,j}$ denote the number of patients in subgroup ℓ which are treated at dose level $d_{j}$ (j=1,…,r). Following the literature [6, 17, 19, 22, 23] we assume that the dose response relationships in each subgroup can be described by a (possibly nonlinear) parametric dose response model, say $\mu_{\ell }(\cdot ,\beta_{\ell }):𝒟\to \mathbb{R}$ with a $\gamma_{\ell }$‐dimensional parameter $\beta_{\ell }$. We model the response of the ith patient treated with dose $d_{j}$ in subgroup ℓ as a normal distributed random variable with variance $\sigma_{\ell }^{2}>0$ and mean $\mu_{\ell }(d_{j},\beta_{\ell })$, that is

(1) $$\begin{array}{l} Y_{\ell ij}=\mu_{\ell }(d_{j},\beta_{\ell })+\epsilon_{\ell ij},i=1,\ldots ,n_{\ell ,j},\;j=1,\ldots ,r,\;\ell =1,\ldots ,k \end{array}$$

where $\epsilon_{\ell ij}$ are independent centered normal distributed errors with variance $\sigma_{\ell }^{2}>0$. This means that we assume the patient responses to be independent. Note that the dose response models $\mu_{\ell }$ and variances $\sigma_{\ell }^{2}$ are allowed to be different for the k different subgroups.

We are interested in testing whether the mean effect in a particular subgroup is similar to the mean effect in the full population. For this purpose we model the mean treatment effect in the full population as a weighted average of the regional treatment effects, where the weights represent the share of each region in the global or overall effect. Similar modelling approaches are employed in the literature [7, 24]. Following these authors we assume that the k subgroup proportions in the full population are known and denote these by $p_{1},\ldots ,p_{k}$, where $p_{\ell }$ represents the positive proportion of the ℓth subgroup in the population ($\sum_{\ell =1}^{k}p_{\ell }=1$). We define an overall (population) effect at dose d by

(2) $$\begin{array}{l} \bar{\mu }(d,\beta ):=\overset{k}{\underset{\ell =1}{\sum }}p_{\ell }\mu_{\ell }(d,\beta_{\ell }) \end{array}$$

where $\beta ={\left(\beta_{1}^{⊤},\ldots ,\beta_{k}^{⊤}\right)}^{⊤}$ denotes the vector of all parameters in the regression models $\mu_{1}.\ldots ,\mu_{k}$ corresponding to the different subgroups. This definition is motivated by the fact that a patient belongs with probability $p_{\ell }$ to the subgroup ℓ, and in this case the expected effect is given by the weighted average $\bar{\mu }=\sum_{\ell =1}^{k}p_{\ell }\mu_{\ell }$. For example, in a multiregional trial $p_{\ell }$ would then denote the proportion of patients in the overall population who belong to region ℓ, with corresponding regional effect $\mu_{\ell }$, and $\bar{\mu }$ the global or overall (weighted) effect across all regions. Note that the overall dose response includes the regional dose response to which it is being compared. As a result, the proportions $p_{1},\ldots ,p_{k}$ significantly influence the test outcomes. For instance, if $p_{1}$ is near 1 for k=2, the overall effect is predominantly driven by region 1. Consequently, the observed maximum deviation in dose response between region 1 and the overall population could be minimal, regardless of the dose response relationship in region 2. In clinical trial practice, the sample size allocation per region is typically planned such that sufficient information to evaluate the drug in its regional context is provided [1].

### 2.1 Assessing Similarity of One Subgroup With the Full Population

With the notations introduced above we can now formalize the problem of testing similarity between the dose response curves of one or more subgroups and the full population. Without loss of generality we assume that the dose response curve of the first subgroup ℓ=1 has to be compared with the full population. We will address this problem by estimating the maximum deviation

(3) $$\begin{array}{l} d_{\infty }:=d_{\infty }(\beta ):=\underset{d\in 𝒟}{\max}|\mu_{1}(d,\beta_{1})-\bar{\mu }(d,\beta )| \end{array}$$

between the (expected) dose response curve $\mu_{1}$ in subgroup 1 and the dose response curve $\bar{\mu }$ defined in (2). Measures of maximum deviation type have found particular attraction to study similarity between curves because they are easy to understand, interpret and to compute. For example, maximum deviation distances of the form (3) are considered to investigate the similarity of curves from two independent groups [15, 16, 18, 20]. In the context of multiregional clinical trials, this distance is used to study differences between the dose response curves of a subgroup and the full population consisting of k=2 subgroups [6]. In order to investigate if the observed dose response relationship of a specific subgroup (here the first one) is sufficiently similar to that of the full population we will develop a test for the hypotheses

(4) $$\begin{array}{l} H_{0}:d_{\infty }\geq \Delta \;\text{versus}\;H_{1}:d_{\infty }<\Delta \end{array}$$

where Δ>0 is a given threshold that depends on the clinical relevance in a particular application. Note that rejecting $H_{0}$ in (4) means to decide that the absolute difference between the dose response curves of the regional subgroup and the full population is smaller than Δ over the whole dose range while keeping the probability for a type 1 error bounded by the significance level, say α.

We are quick in noting that in the special case k=2, the hypotheses in (4) reduce to the hypotheses considered in the literature [6] (up to a constant factor). Note that in this case $p_{2}=1-p_{1}$ and the curve for the full population (2) reduces to $\bar{\mu }(d,\beta )=p_{1}\mu_{1}(d,\beta_{1})+(1-p_{1})\mu_{2}(d,\beta_{2}),$ which yields for the maximum deviation distance in (3) the representation $d_{\infty }=(1-p_{1})\max_{d\in 𝒟}|\mu_{1}(d,\beta_{1})-\mu_{2}(d,\beta_{2})|.$ In this case the alternative hypothesis in (4) coincides with statement (10) in the literature [7].

To estimate the unknown maximal deviation $d_{\infty }$ in (3) we use a plug‐in estimator defined by

(5) $$\begin{array}{l} {\hat{d}}_{\infty }:=d_{\infty }(\hat{\beta })=\underset{d\in 𝒟}{\max}|\mu_{1}(d,{\hat{\beta }}_{1})-\bar{\mu }(d,\hat{\beta })| \end{array}$$

where $\hat{\beta }={\left({\hat{\beta }}_{1}^{⊤},\ldots ,{\hat{\beta }}_{k}^{⊤}\right)}^{⊤}$ and ${\hat{\sigma }}^{2}={\left({\hat{\sigma }}_{1}^{2},\ldots ,{\hat{\sigma }}_{k}^{2}\right)}^{⊤}$ are the maximum likelihood estimators (mle) of the parameters $\;\beta ={\left(\beta_{1}^{⊤},\ldots ,\beta_{k}^{⊤}\right)}^{⊤}$ and $\sigma^{2}={\left(\sigma_{1}^{2},\ldots ,\sigma_{k}^{2}\right)}^{⊤}$, respectively, maximizing the log‐likelihood function

(6) $$\begin{array}{ll} \log(L(\beta ,\sigma^{2})) & =-\overset{k}{\underset{\ell =1}{\sum }}\overset{r}{\underset{j=1}{\sum }}\overset{n_{\ell ,j}}{\underset{i=1}{\sum }}\left\{\log({\left(2\pi \sigma_{\ell }^{2}\right)}^{1/2})\right.\\ & \;\left.+\frac{1}{2\sigma_{\ell }^{2}}{\left(Y_{\ell ij}-\mu_{\ell }(d_{j},\beta_{\ell })\right)}^{2}\right\} \end{array}$$

We reject the null hypothesis in (4) for small values of the statistic ${\hat{d}}_{\infty }$. However, the distribution of ${\hat{d}}_{\infty }$ under the null is complicated and critical values are difficult to obtain (see Theorem 6.3 in the Supporting Information). To address this problem, we propose to use a nonstandard constrained parametric bootstrap test for the hypotheses (4). The pseudo‐code for this procedure is given in Algorithm 1. The most important feature of this Algorithm is that bootstrap data in step (3) is generated using the usual mle $\hat{\beta }$, only if ${\hat{d}}_{\infty }\geq \Delta$ and otherwise a constrained version of the mle $\tilde{\beta }$ defined through

(7) $$\begin{array}{l} \left(\tilde{\beta },{\tilde{\sigma }}^{2})={\text{argmax}}_{\beta :\;d_{\infty }(\beta )=\Delta }L(\beta ,\sigma^{2}\right) \end{array}$$

is employed which boosts the power of the test without violating the asymptotic upper bound α on the type 1 error rate. Note that if bootstrap data is always generated using the unconstrained mle $\hat{\beta }$, changing the decision rule (9) to ${\hat{d}}_{\infty }<{\hat{q}}_{\alpha }^{*}+\epsilon$ also yields an asymptotically valid test, however this test is less powerful.

ALGORITHM 1 **Constrained parametric bootstrap test for hypotheses (4).**

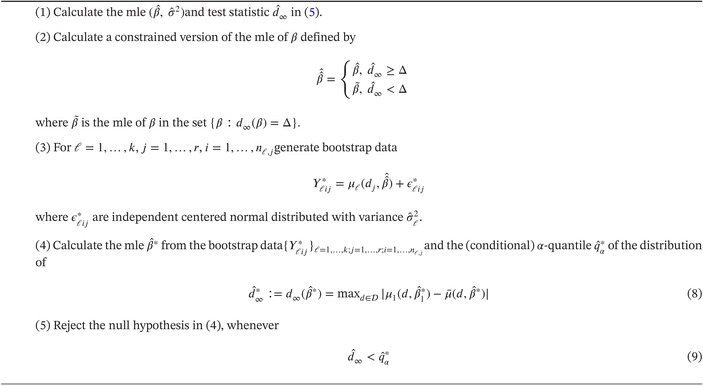

We conclude this section with several remarks. First, we emphasize that in practice the quantile ${\hat{q}}_{\alpha }^{*}$ in Algorithm 1 is simulated by the empirical α‐quantile of B realizations of the bootstrap statistic ${\hat{d}}_{\infty }^{*}$ in (8). More precisely, if ${\hat{d}}_{\infty }^{*,(1)},\ldots ,{\hat{d}}_{\infty }^{*,(B)}$ denote B independent bootstrap copies of ${\hat{d}}_{\infty }^{*}$ generated by Algorithm 1, the empirical α‐quantile of this sample, say ${\hat{q}}_{\alpha }^{*,B}$, is used as an estimate of ${\hat{q}}_{\alpha }^{*}$. In a similar way we can define a p‐value for testing the hypotheses (4) by

(10) $$\begin{array}{l} p_{\infty }:=\frac{1}{B}\overset{B}{\underset{b=1}{\sum }}1\{{\hat{d}}_{\infty }^{*,(b)}\leq {\hat{d}}_{\infty }\} \end{array}$$

Second, we establish in Theorem 6.1 of the Supporting Information the validity of this bootstrap test. For sufficiently large sample sizes the test (9) keeps its nominal level α. To be more precise, consider the set

$$\begin{array}{l} ℰ=\{d\in 𝒟:|\mu_{1}(d,\beta_{1})-\bar{\mu }(d,\beta )|=d_{\infty }\} \end{array}$$

which consists of all dose levels where the (absolute) difference between the two dose response curves is maximal. In most applications the set ℰ has only one element (see Section 3 for some typical nonlinear regression models used in dose response trials) and in such situations the properties of the test can be easily described. If $d_{\infty }>\Delta$ (we call this region *interior of the null hypothesis in* (4)) the probability of rejection converges to 0 for increasing sample sizes. If $d_{\infty }=\Delta$ (we call this region *boundary of the hypotheses in* (4)) the probability of rejection converges to the nominal level α for increasing sample sizes. Moreover, the test (9) detects the alternative with a probability converging to 1 with increasing sample size, which means that it is consistent. A rigorous formulation of this result and a discussion of the case where ℰ consists of more than one point can be found in Theorem 6.1 of the Supporting Information. The finite sample properties of the test are investigated in Section 3.1 by means of a simulation study.

Third, we note that while the literature [6, 7] assesses the similarity between the dose response curves of one subgroup and the full population consisting of only two subgroups, the testing approach proposed in this paper differs even in this simplest case, as follows. The literature [6, 7] considers the size of a “consistency” (or “assurance”) probability $\mathbb{P}({\hat{d}}_{\infty }<\Delta )$ which is calculated under the assumption that the two curves are exactly identical. In particular, the authors do not aim for a control of the probability $\mathbb{P}({\hat{d}}_{\infty }\geq \Delta )$ of a type I error, in contrast to the constrained bootstrap test (9). Consequently, in the case of two groups, their test cannot be compared directly with the one proposed in this paper as it is not calibrated at the correct nominal level. For example, tab. 6 in the literature [7] has to be interpreted with some care as the different tests under consideration are not calibrated for the same type I error rate.

Fourth, we emphasize that we do not compare any treatment groups with the placebo group by testing the hypotheses (4), we only assess whether the dose response relationship of a (regional) subgroup within the trial cohort is similar to the dose response relationship of the overall population. However, the data from the placebo group in the trial is always used for estimating the unknown parameters of the curves and the unknown error variances (see equations (5), (6) and (7)).

Finally, we point out that the hypotheses in (4) are nested. Recalling the definition of the bootstrap in Algorithm 1 it is easy to see that ${\hat{d}}_{\infty ,\Delta_{1}}^{*}\leq {\hat{d}}_{\infty ,\Delta_{2}}^{*}$, where $\Delta_{1}\leq \Delta_{2}$ and ${\hat{d}}_{\infty ,\Delta }^{*}$ denotes the bootstrap statistic (8) calculated by Algorithm 1 for the threshold Δ. Consequently, we obtain for the corresponding quantiles the inequality ${\hat{q}}_{\alpha ,\Delta_{1}}^{*}\leq {\hat{q}}_{\alpha ,\Delta_{2}}^{*}$, and rejecting the null hypothesis in (4) by the test (9) for $\Delta =\Delta_{0}$ also yields rejection of the null for all $\Delta >\Delta_{0}$. Therefore, by the sequential rejection principle, we may simultaneously test the hypotheses in (4) for different Δ≥0 starting at Δ=0 and increasing Δ to find the minimum value ${\hat{\Delta }}_{\alpha }$ for which $H_{0}$ is rejected for the first time. This value could be interpreted as a measure of evidence for similarity with a controlled type I error rate α.

### 2.2 Assessing Similarity of Several Subgroups With the Full Population

In this section we extend the methodology for investigating the similarity of 1≤m≤k subgroups with the full population. Without loss of generality we assume that we are interested in dose response curves corresponding to the subgroups 1,…,m and consider the distance

(11) $$\begin{array}{l} d_{\infty ,\infty }:=d_{\infty ,\infty }(\beta ):=\underset{1\leq i\leq m}{\max}\;\underset{d\in 𝒟}{\max}|\mu_{i}(d,\beta_{i})-\bar{\mu }(d,\beta )| \end{array}$$

In order to establish simultaneously the similarity of the dose response curves in the subgroups 1,…,m with the dose response curve of the full population we consider the null hypothesis

(12) $$\begin{array}{l} H_{0}:d_{\infty ,\infty }\geq \Delta \;\text{versus}\;H_{1}:d_{\infty ,\infty }<\Delta \end{array}$$

for a pre‐specified threshold Δ>0. By the intersection‐union principle [25], it can be tested by applying the test (9) from Section 2.1 for each subgroup i=1,…,m. The null hypothesis (12) is then rejected if and only if all individual tests reject the individual null hypotheses of similarity between the ith curve and the dose response curve of the full population.

However, as tests based on the intersection‐union principle can be conservative, we propose an alternative, more powerful test in the following. To this end, recall the definition of the estimators ${\hat{\beta }}_{1},\ldots ,{\hat{\beta }}_{m}$ and $\hat{\beta }$ in Section 2.1. We estimate $d_{\infty ,\infty }$ by

(13) $$\begin{array}{l} {\hat{d}}_{\infty ,\infty }:=d_{\infty ,\infty }(\hat{\beta })=\underset{1\leq i\leq m}{\max}\;\underset{d\in 𝒟}{\max}|\mu_{i}(d,{\hat{\beta }}_{i})-\bar{\mu }(d,\hat{\beta })| \end{array}$$

and reject the null hypothesis in (12) for small values of ${\hat{d}}_{\infty ,\infty }$. The corresponding quantiles are obtained by the constrained parametric bootstrap test in Algorithm 2, where the decision rule is defined by (15). Similar to Algorithm 1, bootstrap data in step (3) is generated using a constrained version of the maximum likelihood estimator in order to boost power. Only note that in the definition of $\hat{\hat{\beta }}$ the distance $d_{\infty }$ is replaced by the distance $d_{\infty ,\infty }$, since Algorithm 2 targets the hypotheses (12). In practice, the quantile ${\hat{q}}_{\alpha ,\infty }^{*}$ in (15) is estimated by the empirical α‐quantile of the bootstrap sample ${\hat{d}}_{\infty ,\infty }^{*,(1)},\ldots ,{\hat{d}}_{\infty ,\infty }^{*,(B)}$, where for b=1,…,B the quantity ${\hat{d}}_{\infty ,\infty }^{*,(b)}$ is generated by Algorithm 2. The p‐value for testing the hypotheses (12) is defined by

(14) $$\begin{array}{l} p_{\infty ,\infty }:=\frac{1}{B}\overset{B}{\underset{b=1}{\sum }}1\{{\hat{d}}_{\infty ,\infty }^{*,(b)}\leq {\hat{d}}_{\infty ,\infty }\} \end{array}$$

In Section 6.3 of the Supporting Information we show that the decision rule (15) defines a valid test for the hypotheses (12). The finite sample properties of this test are investigated in Section 3.2 by means of a simulation study.

ALGORITHM 2 **Constrained parametric bootstrap test for hypotheses (12).**

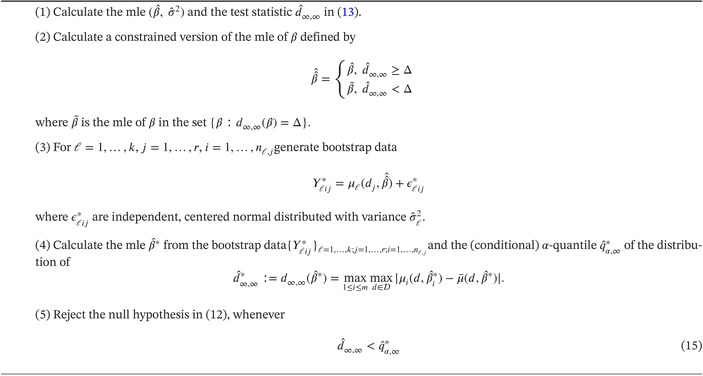

## 3 Finite Sample Properties

In this section we investigate the finite sample performance of the bootstrap procedures defined in Algorithm 1 and Algorithm 2 by means of a simulation study. The constrained maximum likelihood estimator $\tilde{\beta }$ in Step (2) of Algorithm 1 and 2 is computed with the auglag function provided by the alabama package in R and the bootstrap quantile ${\hat{q}}_{\alpha }^{*}$ is determined by B=500 bootstrap replications. To our best knowledge, there do not exist statistical tests for the hypotheses (11), which could be included in such a comparison.

### 3.1 Assessing Similarity of One Subgroup With the Full Population

Our simulation setup is inspired by a multiregional clinical trial design for a Phase II dose finding study for an anti‐anxiety drug [6, 7, 26]. We consider a population with k=3 regional subgroups, where ℓ=1,2,3 denote the Japanese, North American, and European regions, respectively, and assume the proportions $p_{1}=0.1,p_{2}=0.3$ and $p_{3}=0.6$. We test for similarity between the full population and the Japanese subgroup (ℓ=1) using the test (9) in Algorithm 1 for the hypotheses (4). The dose range is 𝒟=[0,150] (in mg) and we consider six dose levels 0, 10, 25, 50, 100 and 150. We fix a total sample size of n=450 patients, but consider two cases for the sample size allocations across subgroups:

(3.1) $$\begin{array}{ll} & n_{1}=150,\;n_{2}=150,\;n_{3}=150 \end{array}$$

(3.2) $$\begin{array}{ll} & n_{1}=66,\;\;n_{2}=192,\;n_{3}=192 \end{array}$$

The motivation for these two scenarios is to investigate a balanced patient allocation in scenario (3.1) and a more realistic allocation in scenario (3.2) where the proportion of Japanese patients in the trial is smaller than those of the two other regions. In both scenarios (3.1) and (3.2) we investigate two choices for the number of patients allocated at each dose level

 
${𝒟}_{=}$: In each subgroup the same number of patients is treated at each dose level 0, 10, 25, 50, 100, and 150.
 
${𝒟}_{\neq }^{\left(1\right)}$: In scenario (3.1) in each subgroup 35,20,20,20,20 and 35 patients are treated at dose levels 0,10,25,50,100 and 150, respectively.
 
${𝒟}_{\neq }^{\left(2\right)}$: In scenario (3.2) in the first subgroup 15, 9, 9, 9, 9 and 15 are treated at dose levels 0,10,25,50,100 and 150, respectively, whereas in the other two subgroups 46, 25, 25, 25, 25 and 46 patients are treated at those dose levels.

The proposed allocation of patients reflects practical settings. In particular, assigning more patients to the placebo and the highest dose groups is sensible, as these groups often provide critical information for assessing both safety and efficacy and, under the monotonicity assumption, lead to the largest expected treatment effects. At the same time, maintaining similar or equal sample sizes across the intermediate dose groups facilitates straightforward comparisons and stabilizes dose–response estimation.

**FIGURE 1:**
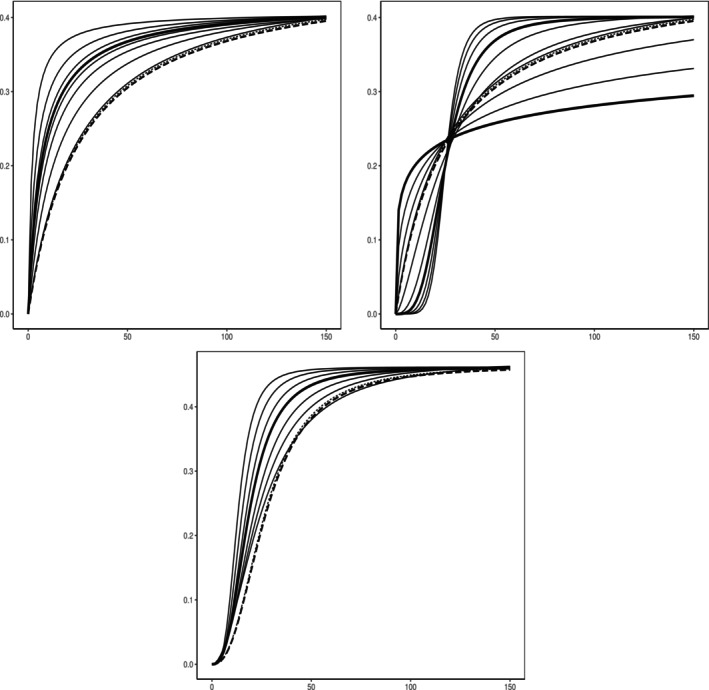
The dose response curves considered in the simulation study. Top row: scenario (A) (left) and scenario (B) (right); bottom row: scenario (C). The (different) solid curves correspond to the first subgroup (for various parameters $E_{\max}$, $ED_{50}$ and h), whereas the dashed and dotted curve correspond to the second and third subgroup, respectively. The thick solid curves satisfy $d_{\infty }=\Delta =0.1$ and mark the boundary of the null hypothesis set.

For the dose response curves in the three subgroups we consider E‐max curves defined by

(3.3) $$\begin{array}{l} E_{0}+\frac{E_{\max}\cdot d^{h}}{d^{h}+ED_{50}^{h}} \end{array}$$

where $E_{0}$ represents the placebo effect of the drug (obtained for d=0), $E_{max}$ denotes the maximum effect, $ED_{50}$ is the dose which produces half of $E_{max}$ and h denotes the slope (or Hill‐) parameter which controls the steepness of the dose response curve. The errors in model (1) are assumed to be centered normal distributed with standard deviation $\sigma_{\ell }=0.1$ (ℓ=1,2,3). For the mean functions we distinguish three scenarios:

$$\begin{array}{ll} \text{(A)}\;\mu_{1}(d) & =\frac{E_{\max}\cdot d}{d+ED_{50}},\mu_{2}(d)=\frac{0.46\cdot d}{d+26},\mu_{3}(d)=\frac{0.46\cdot d}{d+25.5},\\ \text{(B)}\;\mu_{1}(d) & =\frac{E_{max}\cdot d^{h}}{d^{h}+25^{h}},\mu_{2}(d)=\frac{0.46\cdot d}{d+26},\mu_{3}(d)=\frac{0.46\cdot d}{d+25.5},\\ \text{(C)}\;\mu_{1}(d) & =\frac{E_{max}\cdot d^{h}}{d^{h}+ED_{50}^{h}},\mu_{2}(d)=\frac{0.46\cdot d^{2.5}}{d^{2.5}+27^{2.5}},\\ \mu_{3}(d) & =\frac{0.46\cdot d^{2.5}}{d^{2.5}+26.5^{2.5}} \end{array}$$

where the parameters $E_{max},ED_{50}$ and h of the first curve are varied for simulating the rejection probability of the Algorithms in the interior, on the boundary of the null hypothesis and under the alternative. The choice of these parameters is inspired by the literature [6], where the (single) E‐Max candidate dose response model with parameters $E_{0}=0,E_{max}=0.46,ED_{50}=25$ and h=1 is considered. In scenario (A) and (B) the curve for the first subgroup is chosen to deviate more and more from this reference dose response model as we vary the parameters $ED_{50}$, respectively h. Scenario (C) is added to also investigate the case, where both the $ED_{50}$ and Hill‐coefficient of the Japanese curve vary simultaneously. Note that in all three scenarios (A), (B) and (C) the curves for the North American and European subgroup are chosen to be very close to each other as this would generally be expected in practice. To reflect reality, the $E_{max}$ parameter of the Japanese curve is also chosen very similar to the one for the North American and European subgroup (approximately 0.46) throughout all parameter choices. The curves corresponding to the different subgroups are displayed in Figure 1, where we show several curves for the first subgroup.

The rejection probabilities of the test (9) are displayed in Table 1 for scenario (A) and Table 2 for the scenarios (B) and (C), respectively. Note that the $E_{0}$‐coefficients are estimated in all scenarios. We begin with a discussion of the case (A) in Table 1. Here we consider two cases: first, we assume the Hill‐parameters of the curves to be known, resulting in 9 parameters to be estimated overall (three for each curve); second we also estimate these parameters, yielding 12 unknown parameters. The numbers in brackets represent the results for the model in (A) where the Hill‐coefficient is assumed to be known (h=1) and is not estimated. We observe that the test keeps its nominal level α=10% whenever $d_{\infty }\geq \Delta =0.1$ and has reasonable power for $d_{\infty }<\Delta =0.1$. When all four parameters of the models are estimated the test (9) is conservative: even at the boundary $d_{\infty }=\Delta$ the level is smaller than α. On the other hand, if the Hill‐coefficients in all three models are assumed to be known, the simulated level at the boundary is close to α=10%. Fixing the Hill‐parameter yields also a significant improvement in power. For example, under the alternative determined by the Japanese curve with $ED_{50}=10$ and $E_{max}=0.42$ (third row) the power of the test more than doubles in all four dosing scenarios, if we assume the Hill‐coefficients of the curves to be known. For instance, for the equidistant design ${𝒟}_{=}$ the power improves from 0.317 to 0.672. A comparison of the sample size allocations to the different dose levels shows that an equal allocation ${𝒟}_{=}$ yields a more powerful test than the designs ${𝒟}_{\neq }$. Similarly, using the equal sample sizes (3.1) for the populations yields a more powerful test than using a nonuniform design as (3.2).

**TABLE 1:** Scenario (A): Simulated rejection probabilities of the test (9) in Algorithm 1 for subgroup standard deviation $\sigma_{\ell }=0.1$, significance level α=0.1 and equivalence threshold Δ=0.1 for different sample sizes of the subgroups and different dose group sizes.

			Scenario (3.1)		Scenario (3.2)	
	$\left(ED_{50},E_{max}\right)$	$d_{\infty }$	${𝒟}_{=}$	${𝒟}_{\neq }^{\left(1\right)}$	${𝒟}_{=}$	${𝒟}_{\neq }^{\left(2\right)}$
(A)	(25,0.47)	0.00	0.996 (1.000)	0.994 (1.000)	0.954 (0.986)	0.952 (0.996)
	(15,0.44)	0.04	0.884 (0.990)	0.838 (0.972)	0.704 (0.898)	0.670 (0.892)
	(10,0.42)	0.07	0.317 (0.672)	0.278 (0.590)	0.224 (0.450)	0.198 (0.448)
	(8,0.42)	0.09	0.077 (0.228)	0.078 (0.206)	0.090 (0.200)	0.062 (0.192)
	**(7, 0.42)**	0.10	**0.027 (0.094)**	**0.034 (0.082)**	**0.030 (0.092)**	**0.022 (0.098)**
	(6,0.42)	0.11	0.007 (0.018)	0.014 (0.022)	0.014 (0.020)	0.010 (0.046)
	(4,0.41)	0.14	0.000 (0.000)	0.000 (0.002)	0.000 (0.000)	0.002 (0.000)
	(2,0.40)	0.19	0.000 (0.000)	0.000 (0.000)	0.000 (0.000)	0.000 (0.000)

*Note:* Results in brackets are obtained without estimation of the Hill‐parameters of the curves.

**TABLE 2:** Simulated rejection probabilities of the test (9) in Algorithm 1 for subgroup standard deviation $\sigma_{\ell }=0.1$, significance level α=0.1 and equivalence threshold Δ=0.1 for different sample sizes of the subgroups and different dose group sizes.

			Scenario (3.1)		Scenario (3.2)	
	$\left(ED_{50},E_{max},h\right)$	$d_{\infty }$	${𝒟}_{=}$	${𝒟}_{\neq }^{\left(1\right)}$	${𝒟}_{=}$	${𝒟}_{\neq }^{\left(2\right)}$
(B)	**(25, 0.47, 0.3)**	**0.10**	**0.068**	**0.072**	**0.072**	**0.09**
	(25, 0.47, 0.5)	0.06	0.59	0.550	0.420	0.454
	(25, 0.47, 0.75)	0.03	0.958	0.954	0.864	0.848
	(25, 0.47, 1)	0.00	0.994	0.998	0.950	0.944
	(25, 0.43, 1.5)	0.04	0.978	0.944	0.828	0.842
	(25, 0.41, 2.5)	0.08	0.440	0.408	0.322	0.292
	**(25, 0.40, 3.5)**	**0.10**	**0.102**	**0.092**	**0.08**	**0.078**
	(25, 0.40, 4.5)	0.12	0.022	0.036	0.044	0.042
	(25, 0.40, 5.5)	0.13	0.004	0.020	0.018	0.020
	(25, 0.40, 6.5)	0.14	0.000	0.008	0.016	0.022
(C)	(25, 0.47, 2)	0.03	0.966	0.946	0.852	0.782
	(23, 0.47, 2.25)	0.04	0.935	0.850	0.760	0.696
	(21, 0.46, 2.5)	0.06	0.646	0.550	0.394	0.348
	**(18.5, 0.46, 2.75)**	**0.10**	**0.072**	**0.052**	**0.058**	**0.042**
	(17, 0.46, 3)	0.13	0.004	0.008	0.008	0.006
	(15, 0.46, 3.25)	0.16	0.000	0.000	0.000	0.000
	(13, 0.46, 3.5)	0.20	0.000	0.000	0.000	0.000

*Note:* The top and bottom part correspond to scenario (B) and (C), respectively.

Next, we discuss the results for scenario (B) and (C), where all four parameters in the three models are estimated. The rejection probabilities for scenario (B) are displayed in Table 2. Again, the test (9) keeps its nominal level α=10%. Note that in this scenario there are two cases corresponding to the boundary $d_{\infty }=\Delta =0.1$ and the quality of the approximation is different in these cases. In the case h=3.5 and $E_{\max}=0.40$ the approximation of the nominal level at the boundary of the hypotheses is much more accurate as in the case h=0.3 and $E_{\max}=0.47$, in particular for equal sample sizes of the populations (as specified in (3.1)) and a corresponding equidistant design. Similarly to scenario (A) power is improved by equal sizes for the populations and equal sample sizes at the different dose levels. For some cases the results can be compared with the results in Table 1. For example, for h=1.5 and $E_{\max}=0.43$ we obtain $d_{\infty }=0.04$, which corresponds to the case $ED_{50}=15$ and $E_{\max}=0.44$ in Table 1. In this case the alternative in scenario (B) is easier to detect than the alternative in (A), although both cases yield a maximal deviation $d_{\infty }=0.04$. These observations indicate that the power of the test (9) is not completely determined by the distance $d_{\infty }$ but also depends on the properties of the curves. The results in Table 2 for the scenario (C) show a similar picture as for cases (A) and (B) and confirm our findings.

We mention again that, in all three scenarios, the test (generally) performs best if the sample sizes for the subgroups are identical and the patients are allocated uniformly to the different dose levels. These results suggest that, in order to improve the power of the equivalence test (9), the best strategy is to choose the subgroup (and dose group) sizes as uniform as possible. However, we emphasize that this rule of thumb is only applicable, if the variances in all groups and all dose levels are similar.

### 3.2 Assessing Similarity of All Subgroups With a Full Population

In this section we investigate the finite sample performance of the bootstrap procedure defined in Algorithm 2 for the same three scenarios (A), (B), (C) and dose designs defined in Section 3.1. We consider the case where all three subgroups are compared with the full population, that is, m=k=3. The results of the simulation are displayed in Table 3.

**TABLE 3:** Simulated rejection probabilities of the test (15) in Algorithm 2 for subgroup standard deviation $\sigma_{\ell }=0.1$, significance level α=0.1 and equivalence threshold Δ=0.1 for different sample sizes of the subgroups and different dose group sizes.

			Scenario (3.1)		Scenario (3.2)	
	$\left(ED_{50},E_{max},h\right)$	$d_{\infty ,\infty }$	${𝒟}_{=}$	${𝒟}_{\neq }^{\left(1\right)}$	${𝒟}_{=}$	${𝒟}_{\neq }^{\left(2\right)}$
(A)	(25, 0.47, 1)	0.00	0.998	0.994	0.962	0.950
	(15, 0.44, 1)	0.04	0.872	0.832	0.690	0.664
	(10, 0.42,1)	0.07	0.344	0.250	0.258	0.232
	(8, 0.42, 1)	0.09	0.090	0.078	0.072	0.062
	**(7, 0.42, 1)**	**0.10**	**0.030**	**0.038**	**0.022**	**0.028**
	(6, 0.42, 1)	0.12	0.006	0.008	0.006	0.008
	(4, 0.41,1)	0.15	0.000	0.000	0.000	0.000
	(2, 0.40, 1)	0.19	0.000	0.000	0.000	0.000
(B)	**(25, 0.47, 0.3)**	**0.10**	**0.038**	**0.044**	**0.036**	**0.060**
	(25, 0.47, 0.5)	0.06	0.454	0.460	0.384	0.368
	(25, 0.47, 0.75)	0.03	0.930	0.890	0.802	0.762
	(25, 0.47, 1)	0.00	0.992	0.986	0.926	0.936
	(25, 0.43, 1.5)	0.04	0.966	0.922	0.804	0.806
	(25, 0.41, 2.5)	0.08	0.262	0.278	0.212	0.244
	**(25, 0.40, 3.5)**	**0.10**	**0.052**	**0.060**	**0.072**	**0.084**
	(25, 0.40, 4.5)	0.12	0.020	0.008	0.020	0.032
	(25, 0.40, 5.5)	0.13	0.002	0.006	0.008	0.026
	(25, 0.40, 6.5)	0.14	0.002	0.004	0.012	0.016
(C)	(25, 0.47, 2)	0.03	0.952	0.928	0.868	0.802
	(23, 0.47, 2.25)	0.04	0.916	0.850	0.754	0.722
	(21, 0.46, 2.5)	0.06	0.604	0.538	0.462	0.354
	**(18.5, 0.46, 2.75)**	**0.10**	**0.074**	**0.056**	**0.062**	**0.050**
	(17, 0.46, 3)	0.13	0.000	0.004	0.010	0.010
	(15, 0.46, 3.25)	0.16	0.000	0.000	0.000	0.000
	(13, 0.46, 3.5)	0.20	0.000	0.000	0.000	0.000

*Note:* The top, middle and bottom part correspond to scenario (A), (B) and (C), respectively.

The simulation results for Algorithm 2 are in line with its theoretical validity established in Theorem 6.2 of the Supporting Information. That is, the test keeps its nominal level α=10% under the null hypothesis $d_{\infty ,\infty }\geq \Delta =0.1$ and has acceptable power under the alternative hypothesis. Overall, the finite sample performance of Algorithm 2 in the three considered scenarios is very similar to that of Algorithm 1 presented in the previous section. We therefore omit a more detailed discussion for the sake of brevity.

## 4 Numerical Example

In this section we illustrate the proposed methodology analyzing a multiregional dose finding trial example. We apply the methodology to the data from the dose finding study described in section 7 of the literature [27] which was re‐analyzed [18]. The original data set is available in the R package DoseFinding [28]. In this study 369 patients with Irritable Bowel Syndrome (IBS) are investigated at five blinded doses 0 (placebo), 1, 2, 3, and 4 with the primary endpoint being a baseline‐adjusted abdominal pain score. The different dose groups in the study are of approximately equal size.

**FIGURE 2:**
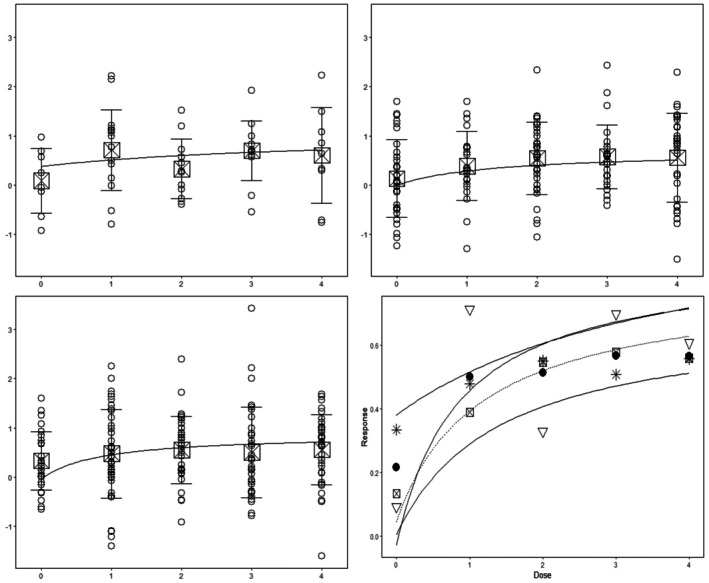
The dose response data and fitted E‐Max curves for the Japanese (top left), North American (top right) and European (bottom left) subgroups in the trial. Circles represent responses, boxes represent the mean of the responses of a given dose group and bars denote 90% confidence intervals for the corresponding means. The bottom right graphic shows the fitted subgroup (solid) and full population (dotted) dose response curves based on the simulated dataset in the case study. Here, the top, middle and bottom solid curve correspond to the Japanese, European and North American subgroup, respectively. Means: ▿ = Japan, ∗ = Europe, ⊠ = North America, • = Population.

In practice, patients can be seen as being randomly recruited from a given population consisting of regional subgroups and then randomized to one of the available treatment groups (i.e., one of the four dose groups or placebo). Since no information about the region was available in the IBS study, we created three regional subgroups by randomly allocating the 369 patients to one of three regional subgroups (i.e., Japan, North America, and Europe) with probabilities $p_{1}=1/7$, $p_{2}=3/7$ and $p_{3}=3/7$, respectively. Accordingly, we also use these proportions in the definition of the overall population dose response function (2). The resulting data set consists of 58 Japanese, 141 North American and 170 European patients. We assume that the subgroup dose response functions are given by three‐parametric E‐Max models as defined in (3.3), where we assume a fixed Hill‐coefficient h=1. The fitted dose response curves of the subgroups based on the maximum likelihood estimates are then given by

$$\begin{array}{ll} \mu_{J}(d) & =0.38+\frac{0.66\cdot d}{d+3.94},\;\mu_{A}(d)=0.00+\frac{0.68\cdot d}{d+1.41}\\ \mu_{E}(d) & =-0.03+\frac{0.90\cdot d}{d+0.85} \end{array}$$

representing the Japanese, North American and European subgroup, respectively. The corresponding AIC values (normalized by the sample sizes) are given by 2.43, 2.49 and 2.55 for the Japanese, North American and European group respectively. These subgroup curves, together with the corresponding dose response data, are displayed in Figure 2 which also shows the estimated effects at the dose levels with corresponding 90% confidence intervals. In particular, the subgroup error variances are estimated as ${\hat{\sigma }}_{J}^{2}=0.58,\;{\hat{\sigma }}_{A}^{2}=0.67$ and ${\hat{\sigma }}_{E}^{2}=0.72$. Figure 2 also presents a more detailed comparison between the three curves and the average population curve.

We now investigate the similarity of subgroup and population dose response curves using Algorithm 1 and Algorithm 2. We use the subscripts “J” (Japan), “A” (North America) and “E” (Europe) to clarify which subgroup is compared to the full population. Throughout this section we fix the similarity threshold as Δ=0.4 and the bootstrap quantiles are calculated by B=1000 bootstrap replications.

First, we test similarity of the European subgroup with the full population by the test (9) in Algorithm 1. The corresponding test statistic, that is, the maximum absolute deviation between the estimated European curve and estimated population curve, is ${\hat{d}}_{\infty ,E}=0.087$. The bootstrap quantile (see step (4) in Algorithm 1) for Δ=0.4 and α=0.05(0.1) is ${\hat{q}}_{\alpha ,E}^{*}=0.086\;(0.113)$. We observe that ${\hat{d}}_{\infty ,E}=0.087>0.086={\hat{q}}_{0.05,E}^{*}$. Thus, the null hypothesis (4) cannot be rejected at significance level α=0.05. However, since ${\hat{d}}_{\infty ,E}=0.087<0.113={\hat{q}}_{0.1,E}^{*}$ we can claim the dose response curves of the European subgroup and the overall population to be similar, that is $d_{\infty ,E}<\Delta =0.4$, at significance level α=0.1. Alternatively, we can compute the corresponding p‐value in (10). By assessing similarity to the European subgroup for Δ=0.4 we obtain $p_{\infty ,E}=0.067$ which is smaller than 0.1 but bigger than 0.05.

Next, we apply the test (9) in Algorithm 1 to assess whether the dose response curves of the Japanese, respectively, North American region and the full population are similar. The corresponding test statistics are given by ${\hat{d}}_{\infty ,J}=0.337$ and ${\hat{d}}_{\infty ,A}=0.116$ and the bootstrap quantiles for the fixed threshold Δ and significance levels α=0.05(0.1) are ${\hat{q}}_{\alpha ,J}^{*}=0.150\;(0.208)$ and ${\hat{q}}_{\alpha ,A}^{*}=0.042\;(0.057)$. Therefore, at both significance levels, we do not claim the dose response relationship of the Japanese and North American subgroup to be similar to the full population, since each test statistic exceeds the corresponding quantile. Again, we come to the same conclusion by calculating the p‐values of the corresponding tests which are given by $p_{\infty ,J}=0.156$ and $p_{\infty ,A}=0.138$.

Whether the subgroups are tested individually with Algorithm 1 or simultaneously with Algorithm 2 depends on the practitioners priorities. Individual tests with Algorithm 1 allow for a more refined understanding of the subgroup similarities as some subgroup dose response curves might be similar to the population curve whereas others are not. However, if Algorithm 1 is applied repeatedly, the significance level α might have to be reduced in each test in order to control the overall type 1 error rate which may yield conservative tests. Applying Algorithm 2 once instead avoids the multiple testing problem on the one hand, but on the other hand only allows investigating if all considered subgroups are similar to the full population.

We now exemplarily test for similarity of all three subgroups with the full population simultaneously using the test (15) in Algorithm 2. The corresponding test statistic is calculated as ${\hat{d}}_{\infty ,\infty }=0.337$ which is the maximum of the three individual test statistics ${\hat{d}}_{\infty ,J},{\hat{d}}_{\infty ,A}$ and ${\hat{d}}_{\infty ,E}$ and coincides with ${\hat{d}}_{\infty ,J}$. The bootstrap quantile for Δ=0.4 (see step (4) of Algorithm 2) is estimated as ${\hat{q}}_{\alpha ,\infty }^{*}=0.189(0.235)$. The p‐value at Δ=0.4 (see (14)) is given by $p_{\infty ,\infty }=0.201$. Hence we do not claim that all subgroup dose response functions are similar to the dose response function of the full population.

**FIGURE 3:**
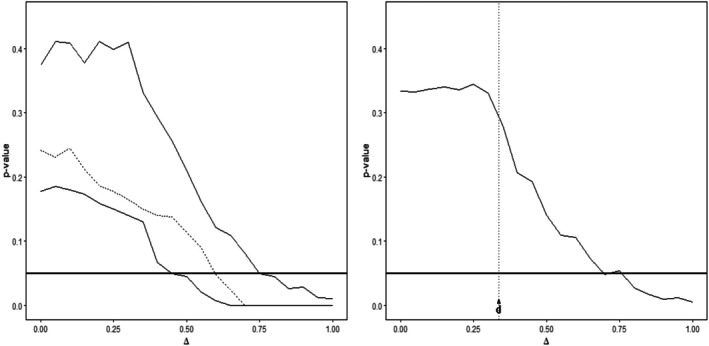
Left panel: p‐values of the test (9) in Algorithm 1 assessing similarity between the Japanese (top solid), North American (dotted) and European (bottom solid) subgroup and the full population for different choices of the threshold Δ. Right panel: p‐values of the test (15) in Algorithm 2 assessing similarity between all three subgroups and the full population for different choices of the threshold Δ. The horizontal solid line marks a *p*‐value of 0.05. The vertical dotted line in the right graphic marks the value of the test statistic ${\hat{d}}_{\infty ,\infty }$.

Figure 3 shows the p‐values of the two tests in relation to the equivalence threshold Δ varying in the interval [0,1]. As one might expect (see also our last remark in Section 2.1), larger thresholds Δ generally yield smaller p‐values and thus a higher chance of rejecting the null hypothesis stating “no similarity” at any given significance level. For example, if Δ is close to one, the p‐values are nearly zero. This illustrates the general fact that, for a large enough threshold Δ, any subgroup dose response curve (s) can be claimed similar to the population curve. However, clearly, the larger the threshold Δ the less meaning any such claim will carry for practical purposes.

Observing Figure 3 one may wonder why the p‐values stay (approximately) constant for thresholds below the corresponding test statistic of the performed test (see, exemplarily, the dotted line in the right panel of Figure 3). We note that this is a consequence of the definition of the constrained mle defined in step (2) of both Algorithms. If the test statistic exceeds the similarity threshold Δ the bootstrap data is always generated from the unconstrained mle which does not depend on Δ. Consequently, the p‐values produced for such thresholds will be (approximately) the same.

## 5 Conclusions and Future Research

In this paper, we develop inference tools for assessing the similarity between dose response curves of one or several subgroups and the full population in multiregional clinical trials. Our approach is based on a statistical test for the null hypothesis that the maximum deviation between the dose response curves is larger than a given threshold Δ. Thus rejection means that the curves of the subgroups deviate by at most Δ from the dose response curve corresponding to the full population over the full dose range. Critical values are determined by a novel parametric bootstrap under a constraint on the parameters such that the null hypothesis is satisfied. We demonstrate by theoretical arguments that our approach defines a valid procedure for similarity testing and by means of a simulation study that it has good finite sample properties. Moreover, the methods are robust with respect to moderate deviations from the model assumptions (these results are not displayed for the sake of brevity).

An essential step of our approach is the specification of the threshold Δ, which has to be carefully discussed for each application with the clinical team. Alternatively, our approach can be used to define a measure of evidence for similarity with a controlled type I error rate, as pointed out in our last remark in Section 2.1. In this paper we focused on the problem of determining whether the dose response relationships between subgroups and the full population in a multiregional trial are similar. In practice, a test may be performed first whether there exists a dose response relationship for the overall population, before proceeding with testing for similarity of dose response. Our approach can readily be used in such a setting as the hierarchical testing procedure will control the overall type I error rate by virtue of the closed testing principle [29, 30]. Likewise, we focus on estimating the dose response relationship of a group of patients based on the data from all dose groups and the placebo group. However, this estimation does not involve the pairwise comparisons between the dose groups and placebo; this is a separate investigation. The reason for this is because we focus on early‐phase dose finding trials, as opposed to the pairwise dose‐placebo comparison typically required in late‐stage, confirmatory trials. If the latter are of interest, then an extension of our approach using the closed testing principle similar to [31] might be considered.

Testing for similarity of dose response in multiregional clinical trials can meaningfully inform the design or conduct of subsequent confirmatory MRCTs. While the number of participating regions in early‐phase trials is often limited, in practice, regions that are key for later submissions are increasingly included at such early development stages. Demonstrating similarity of the dose response relationship across these regions therefore becomes an important objective of such trials, as also indicated in Section 1. When similarity of the dose response relationship cannot be demonstrated across regions, this finding is itself informative. It suggests that in at least one region, the dose response relationship differs meaningfully from that in other regions. This, in turn, implies that the target doses of interest—those to be carried forward into late‐stage confirmatory trials and eventually into market authorization—may need to be different in that region. Thus, even when heterogeneity is observed, the results of early‐phase MRCTs can meaningfully guide the design and conduct of subsequent confirmatory MRCTs. Ultimately, it becomes a sponsor's business and regulatory strategy decision how to best incorporate such information into the planning of confirmatory studies.

Our main focus in this paper is on the maximum deviation between the curves, which makes the definition and interpretation of the threshold relatively easy. However, other measures might also be considered, and an interesting problem of future research is to extend our approach to other metrics, such as the $L_{1}$‐distance, which measures the area between the curves [32, 33]. A further important direction of future research is to extend our approach to situations, where the proportions $p_{\ell }$ of the subgroups are not known (as assumed in this paper) and have to be estimated from the data. In this case it is not reasonable to work with deterministic sample sizes $n_{\ell }$ for the subgroups and patients have to be randomly selected from the full population for the trial. We expect that the methodology can be extended to such situations. Finally, in contrast to other publications on dose finding in multiregional trials, we did not include a model‐selection step via, for example, MCP‐Mod [22] in our methodology, but worked with pre‐selected dose response models for the subgroups from the beginning. We also look forward to generalize our results to account for model uncertainty in the future.

## Conflicts of Interest

The authors declare no conflicts of interest.

## Supporting information


**Data S1.** Supporting Information.

## Data Availability

The data used in the numerical example is openly available in DoseFinding at https://cran.r‐project.org/web/packages/DoseFinding/index.html. Software in R is available from the authors upon request.
