# Supplementary material for: Testing for Similarity of Dose Response in Multiregional Clinical Trials
Source: Stat Med. 2025 Sep 8;44(20-22):e70255. doi: 10.1002/sim.70255 (PMC12417559; doi:10.1002/sim.70255)
Supplement: Supplementary file 1 — Data S1. Supporting Information. [file SIM-44-0-s001.pdf]

6 | SUPPLEMENTARY MATERIAL

6.1 | Basic assumptions and main statements

In this section we provide details about the validity of the two tests (9) and (15) for the hypotheses (4) and (12). We begin stating several assumptions that are required for all theoretical results in this paper.

Assumption 1: The k dose response functions $\mu_1(\cdot,\beta_1),\dots,\mu_k(\cdot,\beta_k)$ depend on unknown parameter vectors $\beta_\ell \in \mathbb{R}^{\gamma_\ell}$, $\ell = 1,\dots,k$. We define $\gamma = \sum_{\ell=1}^k \gamma_\ell$ and summarize these unknown vectors in a single vector $\beta = (\beta_1^\top, ..., \beta_k^\top)^\top$ which belongs to the compact parameter space $\mathcal{B} \subseteq \mathbb{R}^\gamma$.

Assumption 2: For $\ell = 1,\dots,k$ the subgroup dose response function $\mu_\ell(d,\beta_\ell)$ is three times continuously differentiable with respect to β_ℓ and d.

Assumption 3: For $\ell = 1,\dots,k$ we have $n_\ell/n \to \kappa_\ell \in (0,1)$ and $n_{\ell,j}/n_\ell \to \kappa_{\ell,j} \in (0,1)$ for $j = 1,\dots,r$ as $n_\ell \to \infty$.

For a statement of our first main result we introduce the random variable

$$T := \max\Big\{\max_{d\in\mathcal{E}^+} G(d),\ \max_{d\in\mathcal{E}^-} -G(d)\Big\}, \tag{3.4}$$

where $\mathcal{E}^\pm := \{d \in \mathcal{D} : \mu_1(d,\beta_1) - \bar{\mu}(d,\beta) = \pm d_\infty\}$, the process $G = \{G(d)\}_{d\in\mathcal{D}}$ is defined by

$$G(d) := \Big(\tfrac{\partial(\mu_1(d,b_1)-\bar{\mu}(d,b))}{\partial b}\Big)^T\Big|_{b=\beta} Z, \quad d \in \mathcal{D} \tag{3.5}$$

and Z is a centered γ-dimensional normal distributed random variable with block diagonal covariance matrix

$$\Sigma = \mathrm{diag}(\tfrac{1}{\kappa_1}\Sigma_1^{-1},\dots,\tfrac{1}{\kappa_k}\Sigma_k^{-1}) \in \mathbb{R}^{\gamma\times\gamma}, \tag{3.6}$$

where for $\ell = 1,\dots,k$

$$\Sigma_\ell = \frac{1}{\sigma_\ell^2}\sum_{j=1}^r \kappa_{\ell,j}\Big(\frac{\partial}{\partial b_\ell}\mu_\ell(d_j,b_\ell)\Big|_{b_\ell=\beta_\ell}\Big)\Big(\frac{\partial}{\partial b_\ell}\mu_\ell(d_j,b_\ell)\Big|_{b_\ell=\beta_\ell}\Big)^\top \in \mathbb{R}^{\gamma_\ell\times\gamma_\ell}.$$

Theorem 6.1. *Let Assumptions 1-3 be satisfied and assume that the random variable T in* (3.4) *has a continuous distribution function. Furthermore, let $\alpha \in (0,1)$ be small enough, such that the α-quantile of T is negative. Then the test defined by* (9) *for the hypotheses* (4) *is consistent and has asymptotic level α. More precisely,*

(1) if the null hypothesis in (4) *is satisfied, then we have*

$$\limsup_{n\to\infty} \mathbb{P}(\hat{d}_\infty < \hat{q}_\alpha^*) \leq \alpha.$$

(2) if the null hypothesis in (4) *is satisfied and the set*

$$\mathcal{E} = \{d \in \mathcal{D} : |\mu_1(d,\beta_1) - \bar{\mu}(d,\beta)| = d_\infty\}$$

consists of one point, then we have

$$\lim_{n\to\infty} \mathbb{P}(\hat{d}_\infty < \hat{q}_\alpha^*) = \begin{cases} 0, & d_\infty > \Delta, \\ \alpha, & d_\infty = \Delta. \end{cases}$$

(3) if the alternative hypothesis in (4) *is satisfied, then we have*

$$\lim_{n\to\infty} \mathbb{P}(\hat{d}_\infty < \hat{q}_\alpha^*) = 1.$$

Next, we establish a similar result for the test (15). For this purpose we introduce the random variable

$$S := \max\Big\{\max_{(i,d)\in\tilde{\mathcal{E}}^+} G((i,d)),\ \max_{(i,d)\in\tilde{\mathcal{E}}^-} -G((i,d))\Big\}, \tag{3.7}$$

where

$$\tilde{\mathcal{E}}^{\pm} := \{(i,d) \in \{1,...,m\} \times \mathcal{D} : \mu_i(d,\beta_i) - \bar{\mu}(d,\beta) = \pm d_{\infty,\infty}\},$$

the process $G = \{G((i,d))\}_{(i,d)\in\{1,...,m\}\times\mathcal{D}}$ is defined by

$$G((i,d)) := \left(\tfrac{\partial(\mu_i(d,b_i)-\bar{\mu}(d,b))}{\partial b}\right)^T\Big|_{b=\beta} Z, \quad i \in \{1,...,m\},\ d \in \mathcal{D}$$

and Z is the centered γ-dimensional normal distributed random variable defined in (3.5).

Theorem 6.2. *Let Assumptions 1-3 be satisfied and assume that the random variable S in* (3.7) *has a continuous distribution function. Furthermore, let $\alpha \in (0,1)$ be small enough, such that the α-quantile of S is negative. Then the test defined by* (15) *for the hypotheses* (12) *is consistent and has asymptotic level α. More precisely,*

(1) if the null hypothesis in (12) *is satisfied, then we have*

$$\limsup_{n\to\infty} \mathbb{P}(\hat{d}_{\infty,\infty} < \hat{q}^*_{\alpha,\infty}) \leq \alpha.$$

(2) if the null hypothesis in (12) *is satisfied and the set*

$$\tilde{\mathcal{E}} = \{(i,d) \in \{1,...,m\} \times \mathcal{D} : |\mu_i(d,\beta_i) - \bar{\mu}(d,\beta)| = d_{\infty,\infty}\}$$

consists of one point, then we have

$$\lim_{n\to\infty} \mathbb{P}(\hat{d}_{\infty,\infty} < \hat{q}^*_{\alpha,\infty}) = \begin{cases} 0, & d_{\infty,\infty} > \Delta, \\ \alpha, & d_{\infty,\infty} = \Delta. \end{cases}$$

(3) if the alternative hypothesis in (12) *is satisfied, then we have*

$$\lim_{n\to\infty} \mathbb{P}(\hat{d}_{\infty,\infty} < \hat{q}^*_{\alpha,\infty}) = 1.$$

6.2 | Proof of Theorem 6.1

6.2.1 | A preliminary result

We begin with a preliminary result, which is the basic ingredient for the proof of Theorem 6.1.

Theorem 6.3. *Let Assumptions 1-3 be satisfied. Then, as $n_1,\ldots,n_k \to \infty$, the statistic $\hat{d}_\infty$ defined in* (5) *satisfies*

$$\sqrt{n}(\hat{d}_\infty - d_\infty) \xrightarrow{d} T,$$

where the random variable T is defined in (3.4).

Proof. The proof is conducted in four steps. First, observe that under Assumptions 1-3 the maximum likelihood estimate $\hat{\beta}$ defined in (6) satisfies $\sqrt{n}(\hat{\beta} - \beta) \xrightarrow{d} Z$, where $Z \sim \mathcal{N}_\gamma(0,\Sigma)$ with covariance matrix Σ defined by (3.6).

Second, similar arguments as given for the proof of the process convergence in equation (A.7) of the literature[18] show the weak convergence of the process

$$\sqrt{n}\Big\{\big(\mu_1(d,\hat{\beta}_1) - \bar{\mu}(d,\hat{\beta})\big) - \big(\mu_1(d,\beta_1) - \bar{\mu}(d,\beta)\big)\Big\}_{d\in\mathcal{D}} \xrightarrow{d} \{G(d)\}_{d\in\mathcal{D}}$$

in the space $\ell^\infty(\mathcal{D})$ of bounded real-valued functions on $\mathcal{D}$ equipped with the supremum-norm $\|\cdot\|_\infty$, where G is the Gaussian process defined in (3.5). Third, note that the mapping

$$\|\cdot\|_\infty : \begin{cases} \ell^\infty(\mathcal{D}) & \to \mathbb{R}, \\ g & \to \|g\|_\infty = \sup_{d\in\mathcal{D}} |g(x)| \end{cases}$$

is directionally Hadamard differentiable with respect to $(\ell^\infty(\mathcal{D}), \|\cdot\|_\infty)$ with directional Hadamard derivative at $g_0 \in \ell^\infty(\mathcal{D})$ given by

$$D_{g_0}: \begin{cases} \ell^\infty(\mathcal{D}) & \to \mathbb{R}, \\ g & \to D_{g_0}(g) = \max\{\max_{d\in\mathcal{E}^+} g(d), \max_{d\in\mathcal{E}^-} -g(d)\} \end{cases}, \tag{3.8}$$

where $\mathcal{E}^\pm := \{d \in \mathcal{D} : g_0(d) = \pm\|g_0\|_\infty\}$ (see Theorem 2.1 [34]). Fourth, we apply the delta method for directionally Hadamard differentiable functions (see Theorem 2.1 [35]) to complete the proof. □

6.2.2 | Proof of Theorem 6.1

Let $\mathcal{Y} = \{Y_{\ell ij} | i = 1, \ldots, n_{\ell,j}, j = 1, \ldots, r, \ell = 1, \ldots, k\}$ denote the data and define the functions

$$\begin{aligned} g(d) &:= \mu_1(d, \beta_1) - \bar{\mu}(d, \beta), \\ \hat{\hat{g}}(d) &:= \mu_1(d, \hat{\hat{\beta}}_1) - \bar{\mu}(d, \hat{\hat{\beta}}), \\ \hat{g}^*(d) &:= \mu_1(d, \hat{\beta}_1^*) - \bar{\mu}(d, \hat{\beta}^*) \end{aligned}$$

and the corresponding maximum deviations $\hat{d}_\infty^* := \|\hat{g}^*\|_\infty$ and $\hat{\hat{d}}_\infty := \|\hat{\hat{g}}\|_\infty$. Similar arguments as given for the proof of (A.25) in the literature [18] yield the process convergence

$$\sqrt{n}(\hat{g}^* - \hat{\hat{g}}) \xrightarrow{d} \{G(d)\}_{d\in\mathcal{D}}$$

conditionally on $\mathcal{Y}$ in probability which implies

$$\tilde{T}_n^* := D_g(\sqrt{n}(\hat{g}^* - \hat{\hat{g}})) \xrightarrow{d} D_g(G) \stackrel{d}{=} T, \tag{3.9}$$

by the continuous mapping theorem, where D_g denotes the directional Hadamard derivative (3.8) at the function g. Moreover, we have

$$\begin{aligned} \sqrt{n}(\hat{\hat{d}}_\infty - d_\infty) &= D_g(\sqrt{n}(\hat{\hat{g}} - g)) + o_{\mathbb{P}}(1), \\ \sqrt{n}(\hat{d}_\infty^* - d_\infty) &= D_g(\sqrt{n}(\hat{g}^* - g)) + o_{\mathbb{P}}(1). \end{aligned}$$

Then, subtracting the first equation from the second and using sub-additivity of the directional Hadamard derivative yields

$$\sqrt{n}(\hat{d}_\infty^* - \hat{\hat{d}}_\infty) \le \tilde{T}_n^* + o_{\mathbb{P}}(1), \tag{3.10}$$

where there is equality if the set $\mathcal{E}$ consists of a single point (note that in this case the directional Hadamard derivative is linear). Building on these results we can derive part (1) and (2) of Theorem 6.1. In the case $d_\infty > \Delta$, note that

$$\mathbb{P}(\hat{d}_\infty < \hat{q}_\alpha^*) = \mathbb{P}(\hat{d}_\infty < \hat{q}_\alpha^*,\ \hat{d}_\infty \ge \Delta) + \mathbb{P}(\hat{d}_\infty < \hat{q}_\alpha^*,\ \hat{d}_\infty < \Delta),$$

where the first probability converges to zero, which follows by similar arguments as given on page 727 in the literature [18]. The second term converges to zero, since, by Theorem 6.3, $\hat{d}_\infty \xrightarrow{\mathbb{P}} d_\infty > \Delta$. In the case $d_\infty = \Delta$, note that

$$\mathbb{P}(\hat{d}_\infty < \hat{q}_\alpha^*) = \mathbb{P}(\hat{d}_\infty < \hat{q}_\alpha^*,\ \hat{\hat{d}}_\infty = \Delta) + \mathbb{P}(\hat{d}_\infty < \hat{q}_\alpha^*,\ \hat{\hat{d}}_\infty > \Delta),$$

where the first probability sequence is asymptotically bounded above by (or equal to) α, because of (3.9) and (3.10) and the second probability sequence converges to zero, since $q_\alpha < 0$. Finally, in the case $d_\infty < \Delta$ statement (3) follows by the same arguments as given for the proof of (3.18) in Theorem 2 [18].

6.3 | Proof of Theorem 6.2

The proof is analogous to the proof of Theorem 6.1 and only needs some additional adjustments. We start by deriving the asymptotic error distribution of the estimator $\hat{d}_{\infty,\infty}$.

6.3.1 | A preliminary result

Theorem 6.4. *Let Assumptions 1-3 be satisfied. Then, as $n_1, \dots, n_k \to \infty$, the statistic $\hat{d}_{\infty,\infty}$ defined in* (11) *satisfies*

$$\sqrt{n}(\hat{d}_{\infty,\infty} - d_{\infty,\infty}) \xrightarrow{d} S,$$

where the random variable S is defined in (3.7).

Proof. We can copy the proof of Theorem 6.3 and only need to change the second and third step, the first and fourth step can be left unchanged. To this end, only note that by the functional delta method

$$\sqrt{n}\Big\{\big(\mu_i(d,\hat{\beta}_i) - \bar{\mu}(d,\hat{\beta})\big) - \big(\mu_i(d,\beta_i) - \bar{\mu}(d,\beta)\big)\Big\}_{(i,d)\in\tilde{\mathcal{D}}} \xrightarrow{d} \{G((i,d))\}_{(i,d)\in\tilde{\mathcal{D}}}$$

holds true in the space $\ell^\infty(\tilde{\mathcal{D}})$ of bounded real-valued functions on $\tilde{\mathcal{D}} := \{1,...,m\} \times \mathcal{D}$ equipped with the supremum-norm

$$\|g\|_\infty = \sup_{(i,d)\in\{1,...,m\}\times\mathcal{D}} |g(i,d)|$$

and that the supremum-norm is directionally Hadamard differentiable on the space $\ell^\infty(\{1,...,m\} \times \mathcal{D})$ with derivative at g_0 given by

$$D_{g_0} : \begin{cases} \ell^\infty(\tilde{\mathcal{D}}) & \to \mathbb{R}, \\ g & \to D_{g_0}(g) = \max\{\max_{(i,d)\in\mathcal{E}^+} g(i,d),\ \max_{(i,d)\in\mathcal{E}^-} -g(i,d)\} \end{cases},$$

where $\mathcal{E}^\pm := \{(i,d) \in \{1,...,m\} \times \mathcal{D} : g_0(i,d) = \pm\|g_0\|_\infty\}$. □

6.3.2 | Proof of Theorem 6.2

Defining the processes $g((i,d)) := \mu_i(d,\beta_i) - \bar{\mu}(d,\beta)$, $\hat{\hat{g}}((i,d)) := \mu_i(d,\hat{\hat{\beta}}_i) - \bar{\mu}(d,\hat{\hat{\beta}})$, $\hat{g}^*((i,d)) := \mu_i(d,\hat{\beta}_i^*) - \bar{\mu}(d,\hat{\beta}^*)$ as well as $\hat{d}^*_{\infty,\infty} := \|\hat{g}^*\|_\infty$ and $\hat{\hat{d}}_{\infty,\infty} := \|\hat{\hat{g}}\|_\infty$ over the set $\{1,...,m\} \times \mathcal{D}$, we can use the same arguments as given in the proof of Theorem 6.1, where we employ Theorem 6.4 instead of Theorem 6.3.
